# Editorial: Machine learning approaches to antimicrobials: discovery and resistance

**DOI:** 10.3389/fbinf.2024.1458237

**Published:** 2024-08-09

**Authors:** Shira L. Broschat, Shirley W. I. Siu, Cesar de la Fuente-Nunez

**Affiliations:** ^1^ School of Electrical Engineering and Computer Science, Washington State University, Pullman, WA, United States; ^2^ Department of Veterinary Microbiology and Pathology, Washington State University, Pullman, WA, United States; ^3^ Paul G. Allen School for Global Health, Washington State University, Pullman, WA, United States; ^4^ Centre for Artificial Intelligence Driven Drug Discovery, Macao Polytechnic University, Macao, Macao SAR, China; ^5^ Faculty of Applied Sciences, Macao Polytechnic University, Macao, Macao SAR, China; ^6^ Machine Biology Group, Departments of Psychiatry and Microbiology, Institute for Biomedical Informatics, Institute for Translational Medicine and Therapeutics, Perelman School of Medicine, University of Pennsylvania, Philadelphia, PA, United States; ^7^ Department of Bioengineering, University of Pennsylvania, Philadelphia, PA, United States; ^8^ Department of Chemical and Biomolecular Engineering, University of Pennsylvania, Philadelphia, PA, United States; ^9^ Department of Chemistry, University of Pennsylvania, Philadelphia, PA, United States; ^10^ Penn Institute for Computational Science, University of Pennsylvania, Philadelphia, PA, United States

**Keywords:** machine learning, deep learning, antimicrobial resistance, antimicrobial peptides, bacteriocins, WGS, infectious diseases

In 1900, infectious diseases were responsible for nearly 60% of deaths in the United States. By 2010, this had dropped to only 1.62% (Jones et al., 2012). The reason for this dramatic decrease was the introduction of antibiotics in the mid-20th century. However, antimicrobial resistance (AMR) now jeopardizes this achievement. In fact, AMR is a major public health threat with millions of AMR infections occurring annually, often involving resistance to multiple antibiotics. In the United States, AMR accounts for more than 35,000 deaths each year and over 1.27 million deaths globally (CDC, 2024). Unfortunately, because of the high cost of development and lack of return on investment, large pharmaceutical companies are unwilling to commit to developing new antimicrobials to combat AMR without incentives (Dutescu and Hillier, 2021; WHO, 2023). Reducing the expense and uncertainty associated with conventional methods of drug discovery may serve as a powerful incentive.

Machine learning (ML), a branch of artificial intelligence, can assist in the fight against AMR with the development of new antimicrobials (Torres et al., 2022; Maasch et al., 2023; Wong et al., 2023; Santos-Júnior et al., 2024; Wan et al., 2024). It has been used to facilitate drug discovery for many years (Vamathevan et al., 2019). Antimicrobial peptides (AMPs) can be developed as antibiotics, but conventional methods of discovery are not cost-effective. ML algorithms can be developed to predict AMPs, narrowing the number of possible candidates, which can then be tested experimentally for their efficacy (Wan et al., 2024). ML algorithms can also be used as an alternative approach to sequence similarity to predict AMR genes when they have little similarity to known AMR genes (Chowdhury et al., 2019a; Chowdhury et al., 2019b; Chowdhury et al., 2020). These predictions may then be used to help in the treatment of patients.

This Research Topic, “*Machine Learning Approaches to Antimicrobials: Discovery and Resistance*,” consists of five papers: three original research papers and two technology and code papers. One paper applies ML to drug discovery while the others address AMR, as discussed below.

Bacteriocins are antimicrobial peptides produced by bacteria to kill other bacteria. Interest in them has grown because of their ability to control AMR bacteria and their possible use as both broad- and narrow-spectrum antibiotics (Sugrue et al., 2024). In their paper, Akhter and Miller introduce BPAGS, a web application for use in predicting bacteriocins. They consider several different feature evaluation methods and show that the best results are obtained with features reduced using an alternating decision tree algorithm with a support vector machine model. They achieve an accuracy of 99.11% and an AUC of 0.9984 for their test data.

Antibiotic resistance genes (ARGs) are found in bacteria, but in addition to those that occur naturally or are obtained via horizontal gene transfer, point mutations can occur in the bacterial chromosome which can give rise to AMR. These variants of ARGs (ARGVs) can be difficult to identify. Marini et al. propose an approach for identifying ARGVs called KARGVA, for *k*-mer antibiotic resistance gene variant analyzer, which is an extension of their earlier approach KARGA used to identify ARGs. KARGVA performs well on semi-synthetic data, achieving an accuracy of 99.2% for a base rate change of 1%. KARGVA is open-source and publicly available.

*Helicobacter pylori* is a bacterium that infects the stomach and can cause gastritis, ulcers, and even stomach cancer. Unfortunately, *H. pylori* is becoming resistant to several antibiotics (Boyanova et al., 2023). In their study, Yu et al. perform whole-genome sequencing (WGS) for 52 strains of *H. pylori* obtained from 52 different patients to determine their AMR genotypes. The susceptibility of these strains to five different antibiotics is determined experimentally. The authors then use different ML models, including a support vector machine model, with feature selection to predict AMR for the different genotypes and achieve 100% specificity for resistance to amoxicillin and clarithromycin.

Gao et al. also explore the use of WGS in their exploration of the multi-drug resistant bacterium *Acinetobacter baumannii*, which causes many nosocomial infections. While *A. baumannii* has low virulence, those who are immunocompromised or elderly and infirm are at risk, especially if they are ICU patients or have lengthy hospital stays (Ayoub Moubareck and Hammoudi Halat, 2020). Gao et al. combine *k*-mer features from the WGS data with three different ML algorithms to predict AMR to thirteen different drugs. They achieve an average accuracy of 96% for their test data when they use the top 11-mers.

In the final paper on AMR prediction, López-Cortés et al. turn to deep neural networks (DNNs), with data on bacteria obtained via mass spectrometry, to predict AMR to various antibiotics for three different pathogens: *Escherichia coli*, *Staphylococcus aureus*, and *Klebsiella pneumoniae*. Their DNN model, MSDeepAMR, improves upon previous results obtained, and as they point out, their results for predicting resistance to ciprofloxacin are good for all three bacteria.

The articles presented in this Research Topic demonstrate how ML models can be used with a diversity of data to predict antimicrobial peptides in the form of bacteriocins or to predict AMR to numerous antibiotics for a variety of bacteria. They illustrate how models based on ML can be used to assist in combatting AMR.
